# Dietary vitamin D and gastric cancer risk within the stomach cancer pooling (stop) project

**DOI:** 10.1007/s00394-025-03768-w

**Published:** 2025-08-31

**Authors:** Claudia Santucci, Arianna Natale, Claudio Pelucchi, Rossella Bonzi, Nuno Lunet, Samantha Morais, Jesús Vioque, Sandra González-Palacios, Nuria Aragonés, Gemma Castaño-Vinyals, Reza Malekzadeh, Mohammadreza Pakseresht, Eva Negri, Paolo Boffetta, M. Constanza Camargo, Maria Paula Curado, Zuo-Feng Zhang, Stefania Boccia, Carlo La Vecchia, Marta Rossi

**Affiliations:** 1 Department of Clinical Sciences and Community Health, Department of Excellence 2023-27, University of Milan, Milan, Italy; 2 EPIUnit– Instituto de Saúde Pública da Universidade do Porto, Porto, Portugal; 3 Laboratório Para a Investigação Integrativa e Translacional em Saúde Populacional (ITR), Porto, Portugal; 4 Departamento de Ciências da Saúde Pública e Forenses e Educação Médica, Faculdade de Medicina da Universidade do Porto, Porto, Portugal; 5 Consortium for Biomedical Research in Epidemiology and Public Health (CIBERESP), Madrid, Spain; 6 Instituto de Investigación Sanitaria y Biomédica de Alicante, Universidad Miguel Hernandez (ISABIAL-UMH), Alicante, Spain; 7 Department of Health of Madrid, Cancer Epidemiology Section, Public Health Division, Madrid, Spain; 8 Barcelona Institute for Global Health—ISGlobal, Barcelona, Spain; 9 IMIM (Hospital del Mar Medical Research Institute), Barcelona, Spain; 10 Universitat Pompeu Fabra (UPF), Barcelona, Spain; 11 Digestive Oncology Research Center, Digestive Disease Research Institute, Tehran University of Medical Sciences, Tehran, Iran; 12 Department of Agricultural, Food and Nutritional Sciences, University of Alberta, Edmonton, AB Canada; 13 Nutritional Epidemiology Group, Centre for Epidemiology and Biostatistics, University of Leeds, Leeds, UK; 14 Department of Medical and Surgical Sciences, University of Bologna, Bologna, Italy; 15 Stony Brook Cancer Center, Stony Brook University, Stony Brook, NY USA; 16 Division of Cancer Epidemiology and Genetics, National Cancer Institute, Rockville, MD USA; 17 Centro Internacional de Pesquisa, A. C. Camargo Cancer Center, São Paulo, Brazil; 18 Department of Epidemiology, School of Public Health and Jonsson Comprehensive Cancer Center, UCLA Fielding, Los Angeles, CA USA; 19 Section of Hygiene, University Department of Life Sciences and Public Health, Università Cattolica del Sacro Cuore, Rome, Italy; 20 Department of Woman and Child Health and Public Health, Fondazione Policlinico Universitario A. Gemelli IRCCS, Rome, Italy

**Keywords:** Dietary vitamin D, Epidemiology, Gastric cancer, Pooled analysis

## Abstract

**Purpose:**

The evidence regarding the role of vitamin D on gastric cancer (GC) is controversial. Within the Stomach cancer Pooling (StoP) Project, a global consortium of epidemiological studies on GC, we aimed to evaluate the relationship between dietary vitamin D and GC risk.

**Methods:**

Five case–control studies were included in the analysis, accounting for 1875 cases and 5899 controls. Odds ratios (OR) of GC and the corresponding 95% confidence intervals (CI) for tertiles of vitamin D intake were computed using logistic regression models adjusted for relevant confounders, including energy intake. The pooled ORs were computed using random-effect models.

**Results:**

The pooled OR of GC for the highest compared to the lowest tertile of vitamin D intake was 1.06 (95% CI 0.80–1.39), with a *p* for heterogeneity of 0.019. No significant association was found across strata of sex, age, socioeconomic status, smoking status, alcohol intake, and vegetable and fruit consumption.

**Conclusions:**

Our pooled analysis indicates that there is no association between dietary vitamin D and the risk of GC.

**Supplementary Information:**

The online version contains supplementary material available at 10.1007/s00394-025-03768-w.

## Background

Vitamin D deficiency has been associated with increased cancer mortality [1, 2]. In the UK Biobank, an increase in mortality from gastric cancer (GC) was reported for subjects with vitamin D deficiency. Serum 25-hydroxyvitamin D (25(OH)D) concentration was inversely associated with GC mortality, with a dose-risk relationship [2]. Vitamin D deficiency has also been associated with a higher risk of *Helicobacter pylori* (*Hp*) infection [3], the main recognized GC risk factor.

Vitamin D affects cellular apoptosis and proliferation in GC cells by inhibiting the expression of hedgehog signaling target genes as Gli1 and of the Wnt/β-catenin signaling pathway, and by increasing the expression of BAX and of the acid sphingomyelinase and PTEN [4–7]. Also, in GC tissues the expression of vitamin D receptor is reduced [8]. In orthotopic GC nude mice model, low-dose oral administration or intraperitoneal injection of 1,25-Dihydroxyvitamin D3 suppressed GC growth [7].

A substantial proportion (60–80%) of vitamin D is obtained from sun exposure (depending on latitude, season, skin color, and other factors) and the remaining derives from diet (supplements excluded) [9]. Major dietary sources include oily fish, eggs, meat, and dairy products [10].

Some case–control studies found significant GC risk reduction for higher compared to lower dietary vitamin D levels [11, 12]. However, a meta-analysis reported no association between dietary vitamin D and GC risk [13]. The same meta-analysis found no association also for serum 25(OH)D. Another meta-analysis on serum 25(OH)D, on the other hand, showed significantly lower levels in GC cases than in healthy controls [14]. The issue is, therefore, still open to discussion.

To further evaluate the relation between dietary vitamin D and GC risk, we conducted a pooled analysis of case–control studies within the International “Stomach cancer Pooling (StoP) Project” [15, 16].

## Methods

The StoP Project was established in 2012 as an international collaborative effort with the aim to elucidate determinants of risk and outcome of GC [16]. All the studies participating in the StoP consortium [16] were conducted in accordance with applicable laws, regulations and guidelines for protection of human subjects, and the StoP Project received ethical approval from the University of Milan Review Board (reference no. 19/15 of 01/04/2015). Principal investigators of the studies included in the StoP Project agreed to participate in the consortium by providing a signed data transfer agreement and the original dataset (or part of it) to the pooling center. Questionnaires used for data collection and any other useful information, such as codebooks and labels, were also provided from the participating studies. Data harmonization of core variables, such as those related to sociodemographic and selected lifestyle factors, was carried out at the Coordinating Center in Milan, Italy, according to a pre-specified format. For the present analysis, the version 3.3 of the StoP dataset was used, which includes 34 case–control or nested case–control studies totaling 13,121 GC cases and 31,420 controls [17].

Five studies participating in the StoP Project had information on the intake of vitamin D and were included in the present analysis: one each from Italy [18], Iran [19], and Portugal [20], and two from Spain– Spain 1 [21] and Spain 2 [22]. Dietary habits were assessed using food frequency questionnaires (FFQ), designed to reflect the dietary pattern of each study population. The number of items included in the FFQs ranged from 78 in the Italian study to 140 in the Portuguese study. Vitamin D intake was derived from each study using information from FFQ and specific food composition tables (FCT) [23–27]. We categorized vitamin D intake into tertiles based on the study-specific distribution among controls.

Structured questionnaires were used in each study to collect information on participants’ sociodemographic data, including sex, age, and socioeconomic status, as well as lifestyle characteristics, such as tobacco smoking and alcohol drinking, and family history of GC. Data on *Hp* infection status were available for 937 cases and 3524 controls from three studies: Portugal [20], Spain 1 [21], and Iran [19].

The association between Vitamin D and GC was estimated through a two-stage modeling approach [28]. First, we computed the odds ratios (OR) and the corresponding 95% confidence intervals (95% CI) of GC for tertiles of vitamin D intake in each study using multivariable logistic regression models adjusted for sex, age (in quinquennia), socioeconomic status (low, intermediate, high), smoking status (never, former, current low, intermediate, and high), family history of GC, *Hp* infection serostatus (when available), and total energy intake (using study-specific quartiles). The covariates were selected a priori, based on the current knowledge of GC risk factors and their associations with dietary exposures. For covariates with up to 5% missing values, we replaced missing data by assigning them to the most frequent value; for covariates with a higher proportion, we treated them with an ad hoc category. In the second stage, the pooled effect estimates were computed using random-effect models to take into account the heterogeneity of risk estimates through the DerSimonian and Laird method [29]. This was performed for the comparison of the third tertile of vitamin D intake versus the first one. Heterogeneity between studies was assessed using the χ^2^ test, and inconsistency was measured using the I^2^ statistic, which represents the proportion of total variation attributable to between-study variance [30]. We also conducted stratified analyses according to different study characteristics, including sex, age (< 55, 55–65, > 65 years), socioeconomic status (low, intermediate, high), smoking status (never, former, current), alcohol intake (never, low, intermediate, high), and vegetable and fruit consumption (low, intermediate, high). We examined the funnel plots [31] and used Egger’s test for funnel plot to evaluate asymmetry [32]. Statistical analyses were performed using R-software (version 4.3.2; R Development Core Team, 2024).

## Results

Table 1 shows the distribution of 1875 GC cases and 5899 controls in the five studies included in the analyses, along with the main study characteristics. Three of the five studies were population-based (Iran, Portugal, and Spain 1) and two (Iran, Spain 2) hospital based. The Portuguese and Spain 2 studies had the highest percentage of GC cases (34% and 21%, respectively).

**Table 1:** Distribution of 1875 gastric cancer cases and 5899 controls of each study included in the StoP (Stomach cancer Pooling) Consortium

Study	Study type	Study area	StoP project country	Study period	Cases (%)	Controls (%)	Total (%)
Lucenteforte et al., [18]	Hospital-based	Milan, Italy	Italy	1997–2007	230 (12.3)	547 (9.3)	777 (10.0)
Pakseresht et al., [19]	Population-based	Ardabil, Iran	Iran	2005–2007	284 (15.1)	304 (5.2)	588 (7.6)
Lunet et al., [20]	Population-based	Porto, Portugal	Portugal	1999–2006	633 (33.8)	1600 (27.1)	2233 (28.7)
Castaño-Vinyals et al., [21]	Population-based	10 provinces, Spain	Spain 1	2008–2012	330 (17.6)	2993 (50.7)	3323 (42.7)
Santibanez et al., [22]	Hospital-based	Valencia, Spain	Spain 2	1995–1999	398 (21.2)	455 (7.7)	853 (11.0)
*Total*					1875	5899	7774

**Fig. 1:**
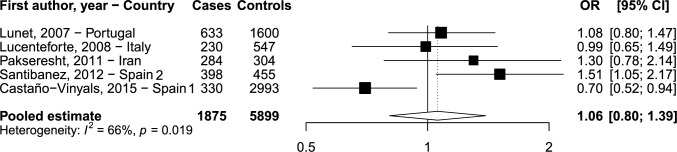
Forest plot for study-specific and pooled odds ratios (OR)^a^ with corresponding 95% confidence intervals (CI) of gastric cancer for the highest versus the lowest tertile of vitamin D intake. ^a^Estimated by two-stage meta-analysis using logistic regression models adjusted for sex, age, socioeconomic status, smoking status, family history of gastric cancer, *Helicobacter pylori* infection (when available), and total energy intake.

Table 2gives the upper cutpoints of study-specific tertiles of vitamin D intake and the ORs and corresponding 95% CI of GC according to tertiles. The OR for the highest versus the lowest tertile of dietary vitamin D was 0.99 (95% CI 0.65–1.49, *p* for trend: 1.000) in the study from Italy, 1.30 (95% CI 0.78–2.14, *P* for trend: 0.301) from Iran, 1.08 (95% CI 0.80–1.47, *p* for trend: 0.536) from Portugal, 0.70 (95% CI 0.52–0.94, *p* for trend: 0.018) in Spain 1 study, and 1.51 (95% CI 1.05–2.17, *p* for trend: 0.025) in Spain 2 study. Figure 1 shows the forest plot for study-specific and pooled OR with corresponding 95% CI of GC for the highest versus the lowest tertile of vitamin D intake. There was significant heterogeneity between studies (I^2^ = 66%, *p*: 0.019) and the pooled OR of GC for the highest vs the lowest tertile of vitamin D was 1.06 (95% CI 0.80–1.39).

**Table 2:** Odds ratios (OR) and corresponding 95% confidence intervals (CI) of gastric cancer according to study-specific tertiles of vitamin D intake

Study	Tertiles of intake				
	I	II	III	*P* for trend	Continuous OR^a^
*Italy*					
Upper cutpoints (μg/day)	2.38	3.32	–		
Cases, n (%)	72 (31.30%)	71 (30.87%)	87 (32.22%)		1.05 (0.89–1.24)
OR^b^ (95% CI)	1	0.84 (0.56–1.28)	0.99 (0.65–1.49)	*1.000*	
*Iran*					
Upper cutpoints (μg/day)	0.99	1.56	–		
Cases, n (%)	101 (35.56%)	100 (35.21%)	83 (29.23%)		1.14 (0.97–1.35)
OR^b^ (95% CI)	1	1.23 (0.79–1.92)	1.30 (0.78–2.14)	*0.301*	
*Portugal*					
Upper cutpoints (μg/day)	2.58	4.04	–		
Cases, n (%)	256 (40.44%)	154 (24.33%)	223 (35.23%)		1.05 (0.94–1.18)
OR^b^ (95% CI)	1	0.71 (0.52–0.97)	1.08 (0.80–1.47)	*0.536*	
*Spain 1*					
Upper cutpoints (μg/day)	2.08	3.16	–		
Cases, n (%)	126 (38.18%)	94 (28.48%)	110 (33.33%)		0.92 (0.82–1.05)
OR^b^ (95% CI)	1	0.71 (0.53–0.96)	0.70 (0.52–0.94)	*0.018*	
*Spain 2*					
Upper cutpoints (μg/day)	3.79	6.08	–		
Cases, n (%)	98 (24.62%)	126 (31.66%)	174 (43.72%)		1.25 (1.09–1.42)
OR^b^ (95% CI)	1	1.18 (0.82–1.70)	1.51 (1.05–2.17)	*0.025*	

^a^Estimated for an increment of intake equal to 1 standard deviation among controls

^b^Adjusted for sex, age, socioeconomic status, smoking status, family history of gastric cancer, *Helicobacter pylori* infection (when available), and total energy intake

Supplementary Figure 1 gives the funnel plot of studies on the ORs of GC risk for the highest versus the lowest tertile of vitamin D intake. Both visual inspection of the funnel plot of studies and Egger’s test (*p*: 0.373) did not indicate asymmetric distribution. Table 3 reports the ORs and the corresponding 95% CI of GC for vitamin D intake tertiles in strata of sex, age, socioeconomic status, smoking status, alcohol intake, and vegetable and fruit intake. No associations were found in any of the analyzed strata. No heterogeneity was detected between strata. Supplementary Figures 2–7 show the forest plots for study-specific and pooled OR with 95% CI of GC for the highest versus the lowest tertile of vitamin D intake, in these strata.

**Table 3:** Pooled odds ratios (OR) and corresponding 95% confidence intervals (CI) of gastric cancer in strata of selected variables according to study-specific tertiles of vitamin D intake

	OR (95% CI) ^a^		
	II vs I	III vs I	*P*-value ^b^
*Sex*			
Male	0.90 (0.67–1.22)	1.06 (0.77–1.47)	0.949
Female	0.84 (0.63–1.13)	1.05 (0.74–1.48)	
*Age (years)*			
< 55	1.00 (0.71–1.42)	1.27 (0.90–1.80)	0.574
55–65	0.91 (0.65–1.27)	1.05 (0.62–1.78)	
> 65	0.92 (0.53–1.58)	1.00 (0.76–1.32)	
*Socioeconomic status*			
Low	0.82 (0.57–1.18)	0.91 (0.64–1.28)	0.632
Intermediate	1.08 (0.77–1.51)	1.24 (0.66–2.32)	
High	0.83 (0.48–1.44)	1.12 (0.65–1.91)	
*Smoking status*			
Never	0.87 (0.67–1.13)	1.08 (0.76–1.52)	0.984
Former	0.79 (0.54–1.16)	1.05 (0.62–1.79)	
Current	1.00 (0.70–1.42)	1.11 (0.78–1.58)	
*Alcohol intake * ^*c*^			
Never	0.91 (0.47–1.78)	0.88 (0.38–2.05)	0.181
Low	0.79 (0.57–1.10)	1.16 (0.61–2.19)	
Intermediate	0.72 (0.53–0.99)	0.81 (0.59–1.12)	
High	0.88 (0.57–1.37)	1.47 (0.95–2.29)	
*Vegetable and fruit consumption*			
Low	0.91 (0.63–1.33)	1.10 (0.77–1.57)	0.436
Intermediate	1.11 (0.75–1.63)	1.41 (0.96–2.06)	
High	0.73 (0.53–1.01)	0.97 (0.62–1.53)	

^a^Estimated by two-stage meta-analysis using logistic regression models adjusted for sex, age, socioeconomic status, smoking status, family history of gastric cancer, *Helicobacter pylori* infection (when available), and total energy intake

^b^*P*-value for heterogeneity between strata (χ^2^) of III vs I tertile

^c^Data from Iran study was not included because of missing data on alcohol intake

## Discussion

Our results do not support the existence of an association between dietary vitamin D and risk of GC. The lack of relationship was consistent across all the strata considered.

Vitamin D deficiency has a recognized impact at public health level, not only for its consequence on bone disease [33, 34], but also for a direct association reported with autoimmune diseases, hypertension, infectious diseases, and selected cancers [2, 3, 14, 35]. In particular, lower serum 25(OH)D levels have been associated with higher GC risk, and in the UK Biobank cohort, the association was significant for 25(OH)D levels below 50 nmol/mL [2], which is considered the minimum sufficient level [36]. Moreover, adequate 25(OH)D levels have been associated with *Hp* eradication [37].

Circulating vitamin D levels derive from multiple factors, mostly sun exposure, with only a small portion coming from diet [9, 10]. Several studies have examined associations between dietary vitamin D and cancer risk [38–40]. Inverse associations of vitamin D with cancers of the digestive tract, including colorectal [41], esophageal and oral/pharyngeal cancer [42] were reported. Concerning GC, a meta-analysis of four case–control studies on a total of 1652 cases, found a pooled OR of 1.09 (95% CI 0.94–1.25) for higher versus lower vitamin D intakes [13], in line with our results. A case–control study conducted in Vietnam on 1182 CG cases and 2995 controls found a significant risk reduction of 32% for the highest versus the lowest quintile of vitamin D intake [11]. That result was consistent across strata of sex, tobacco smoking, and *Hp* infection status. Another study, conducted in Jordan on 173 GC cases and 313 controls, found a 50% reduction in GC risk for the highest versus to the lowest tertile [12].

A major strength of this study is the large sample size, comprising 1875 GC cases (and 5899 controls), which doubles the number of cases reported in the meta-analysis by Khayatzadeh and colleagues [13], adding relevant original data to the existing evidence. Among the limitations, the studies included in our analysis utilized a case–control design, which may be affected by selection and recall bias [45]. However, recall bias has been reported to be limited in case–control studies of micronutrients [46]. Our analysis relied on previously conducted studies using different methodologies, such as the use of diverse FFQs and FCT, possibly contributing to the heterogeneity of vitamin D estimates between studies. Notably, the estimate of vitamin D intake in the Iranian study was considerably lower than in the other studies, and the estimate in the Spain 1 study was lower than that in the Spain 2 study. This can be due to different geographical locations and dietary patterns. In particular, the Spain 1 study was conducted in inland provinces, possibly characterized by a less traditional Mediterranean dietary habits, which have been directly related to serum 25(OH)D [43]. In addition, differences in the estimates of vitamin D intake may be due to the use of different FCTs: the Spanish FCT in the Spain 1 study and the more recent and reliable USDA FCT in the Spain 2 study [23, 24]. However, to compute the pooled estimates, we used vitamin D tertiles based on the study-specific distribution in our two-stage meta-analysis approach. Moreover, our pooled ORs were estimated taking into account the possible confounding effects of several variables, including energy intake and vegetable and fruit consumption. The lack of comprehensive information on *Hp* infection status, the main risk factor for non-cardia GC [44], could be a potential limitation since we were able to adjust for *Hp* infection in studies where the information was available.

We found significant heterogeneity between studies, which was addressed by applying random-effects models. This can be partially attributed to differences of vitamin D estimates between studies. However, when we excluded data from the Iran study, which showed low vitamin D levels, we obtained a pooled OR of 1.03 (95% CI: 0.75–1.40). The only inverse association was observed in the Spain 1 study. This may be due to concerns regarding vitamin D measurement, residual confounding or chance, and should be interpreted with caution. When we excluded data from the Spain 1 study from the main analysis, the pooled OR became 1.19 (95% CI: 0.98–1.45). Moreover, lifestyle factors and population characteristics between studies may alter the effect of vitamin D from diet on GC risk. These include outdoor sunlight exposure, geographic region of residence, body mass index, and genetic differences. Vitamin D from sun exposure can obscure the effect of dietary vitamin D. Populations in countries closer to the equator tend to have higher vitamin D from sun exposure, reducing the impact of diet on serum 25(OH)D levels*.* We were not able to adjust for intensity of sunlight exposure in our study because information on daily sun exposure was lacking and country latitudes of our study participants were similar (range 38–45°). However, the relationship between dietary vitamin D and GC risk did not increase in studies with higher country latitudes [11–13]. In addition, a relationship between dietary vitamin D and GC risk could be confounded by the consumption of some of their food sources, such as salt-preserved fish, cheese and cured meats, which have been positively associated with GC risk [47].

In conclusion, the present analysis carried out by pooling data from five epidemiological studies provides convincing evidence of a null association of dietary vitamin D and GC risk.

## Supplementary Information

Below is the link to the electronic supplementary material.

Supplementary file1 (DOCX 2133 KB)

## Data Availability

The data underlying this article will be shared upon reasonable request to the corresponding author.
