# Supplementary material for: Dietary vitamin D and gastric cancer risk within the stomach cancer pooling (stop) project
Source: Eur J Nutr. 2025 Aug 31;64(6):269. doi: 10.1007/s00394-025-03768-w (PMC12399693; doi:10.1007/s00394-025-03768-w)
Supplement: Supplementary file 1 — Supplementary file1 (DOCX 2133 KB) [file 394_2025_3768_MOESM1_ESM.docx]

**SUPPLEMENTARY MATERIAL**

**Manuscript title:** Dietary vitamin D and gastric cancer risk within the Stomach Cancer Pooling (StoP) Project

**Table of contents**

[**Supplementary Figure 1.** Funnel plot of studies on the odds ratios of gastric cancer risk for the highest versus the lowest tertile of vitamin D intake. (*p* Egger’s test for funnel plot asymmetry=0.3728) 2](#_Toc201154379)

[**Supplementary Figure 2.** Forest plot for study-specific and pooled odds ratios (OR)^a^ with 95% confidence intervals (CI) of gastric cancer for the highest versus the lowest tertile of vitamin D intake, according to sex. 3](#_Toc201154380)

[**Supplementary Figure 3.** Forest plot for study-specific and pooled odds ratios (OR)^a^ with 95% confidence intervals (CI) of gastric cancer for the highest versus the lowest tertile of vitamin D intake, according to age classes. 4](#_Toc201154381)

[**Supplementary Figure 4.** Forest plot for study-specific and pooled odds ratios (OR)^a^ with 95% confidence intervals (CI) of gastric cancer for the highest versus the lowest tertile of vitamin D intake, according to socioeconomic status. 5](#_Toc201154382)

[**Supplementary Figure 5.** Forest plot for study-specific and pooled odds ratios (OR)^a^ with 95% confidence intervals (CI) of gastric cancer for the highest versus the lowest tertile of vitamin D intake, according to smoking status. 6](#_Toc201154383)

[**Supplementary Figure 6.** Forest plot for study-specific and pooled odds ratios (OR)^a^ with 95% confidence intervals (CI) of gastric cancer for the highest versus the lowest tertile of vitamin D intake, according to alcohol consumption. 7](#_Toc201154384)

[**Supplementary Figure 7.** Forest plot for study-specific and pooled odds ratios (OR)^a^ with 95% confidence intervals (CI) of gastric cancer for the highest versus the lowest tertile of vitamin D intake, according to vegetables and fruits intake consumption. 8](#_Toc201154385)

# **Supplementary Figure 1.** Funnel plot of studies on the odds ratios of gastric cancer risk for the highest versus the lowest tertile of vitamin D intake. (*p* Egger’s test for funnel plot asymmetry=0.3728)

# **Supplementary Figure 2.** Forest plot for study-specific and pooled odds ratios (OR)^a^ with 95% confidence intervals (CI) of gastric cancer for the highest versus the lowest tertile of vitamin D intake, according to sex.

**
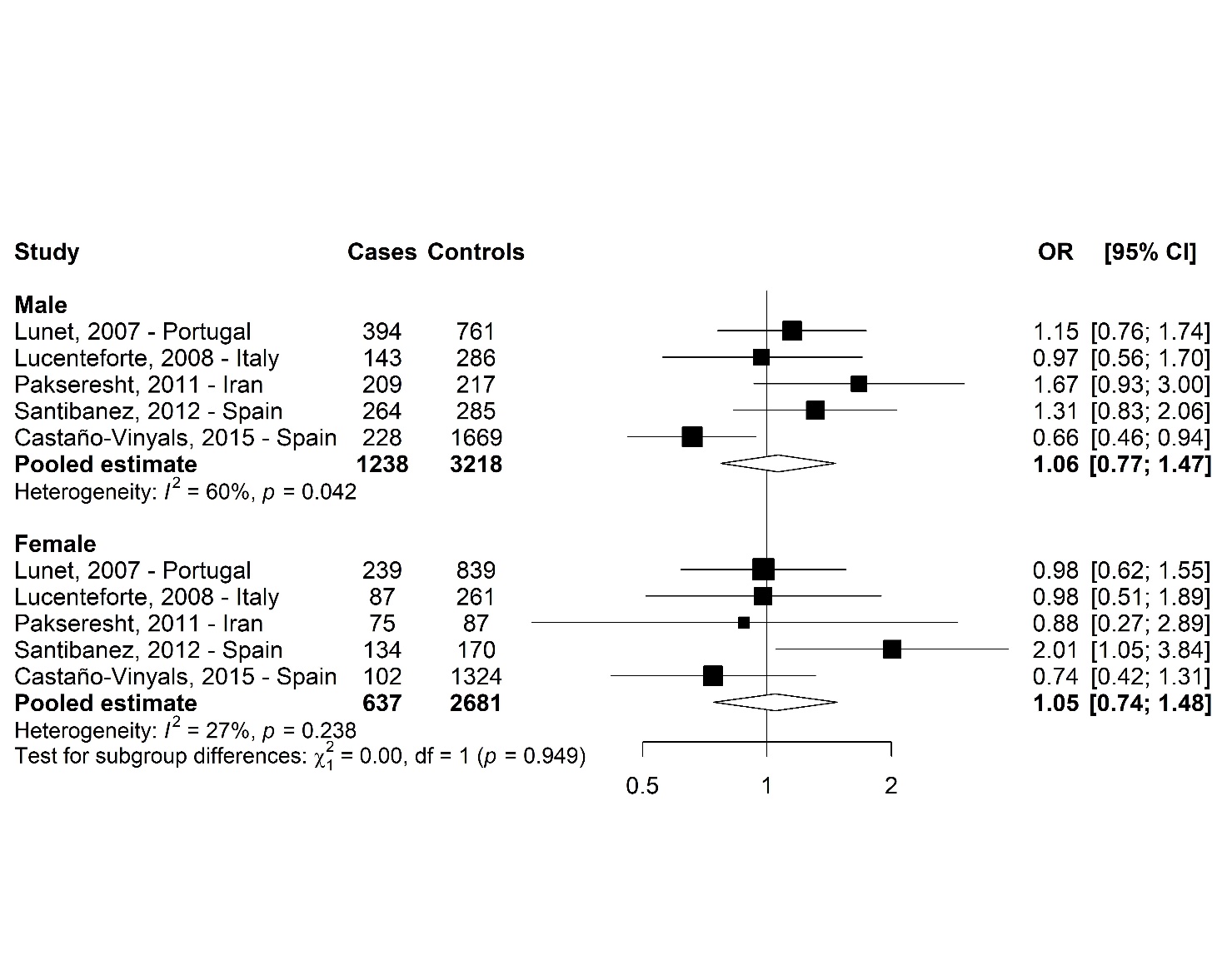
**

^a^ Estimated by two-stage meta-analysis using logistic regression models adjusted for sex, age, socioeconomic status, smoking status, family history of gastric cancer, *Hp* infection (when available), and total energy intake.

# **Supplementary Figure 3.** Forest plot for study-specific and pooled odds ratios (OR)^a^ with 95% confidence intervals (CI) of gastric cancer for the highest versus the lowest tertile of vitamin D intake, according to age classes.

**
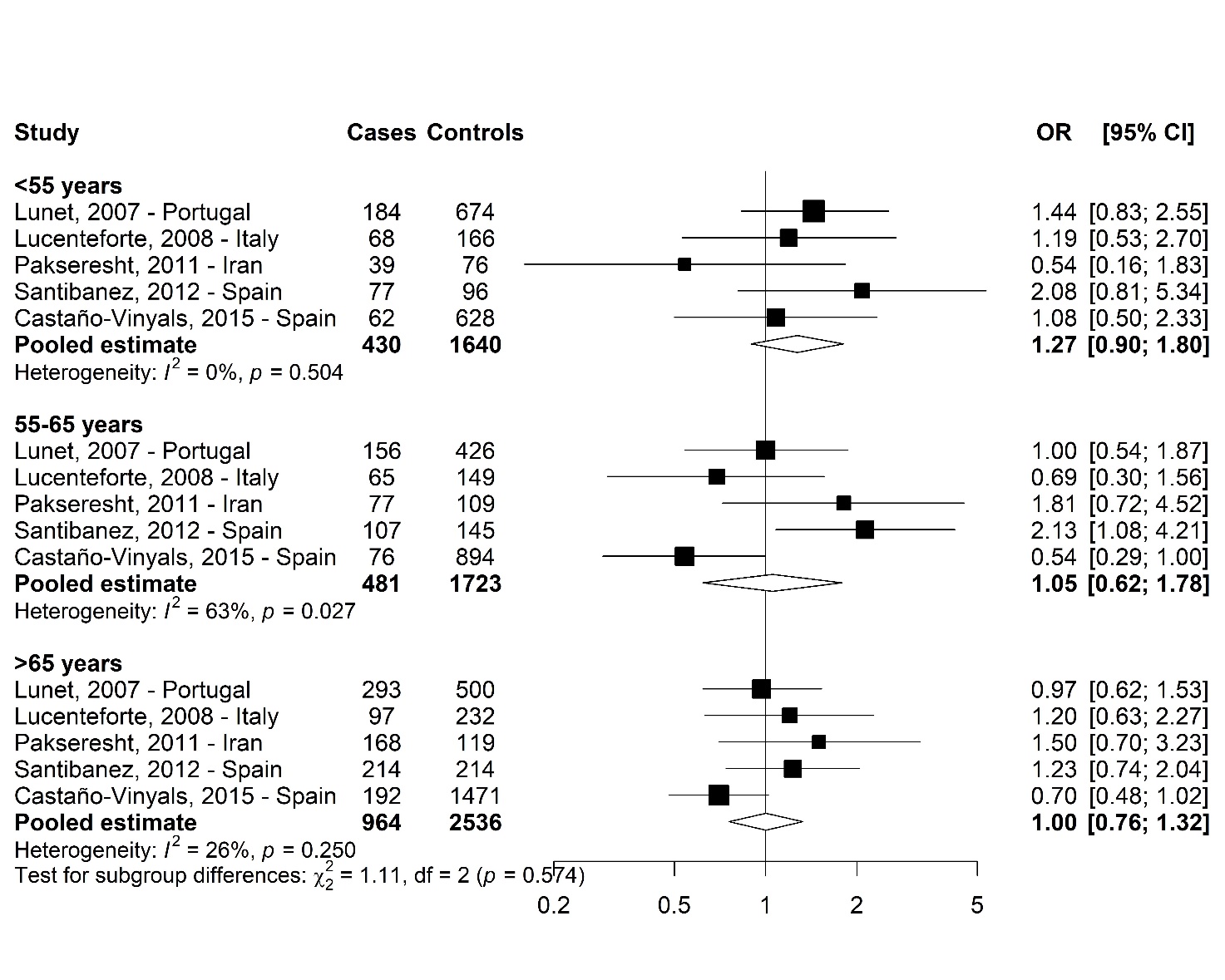
**

^a^ Estimated by two-stage meta-analysis using logistic regression models adjusted for sex, age, socioeconomic status, smoking status, family history of gastric cancer, *Hp* infection (when available), and total energy intake.

# **Supplementary Figure 4.** Forest plot for study-specific and pooled odds ratios (OR)^a^ with 95% confidence intervals (CI) of gastric cancer for the highest versus the lowest tertile of vitamin D intake, according to socioeconomic status.


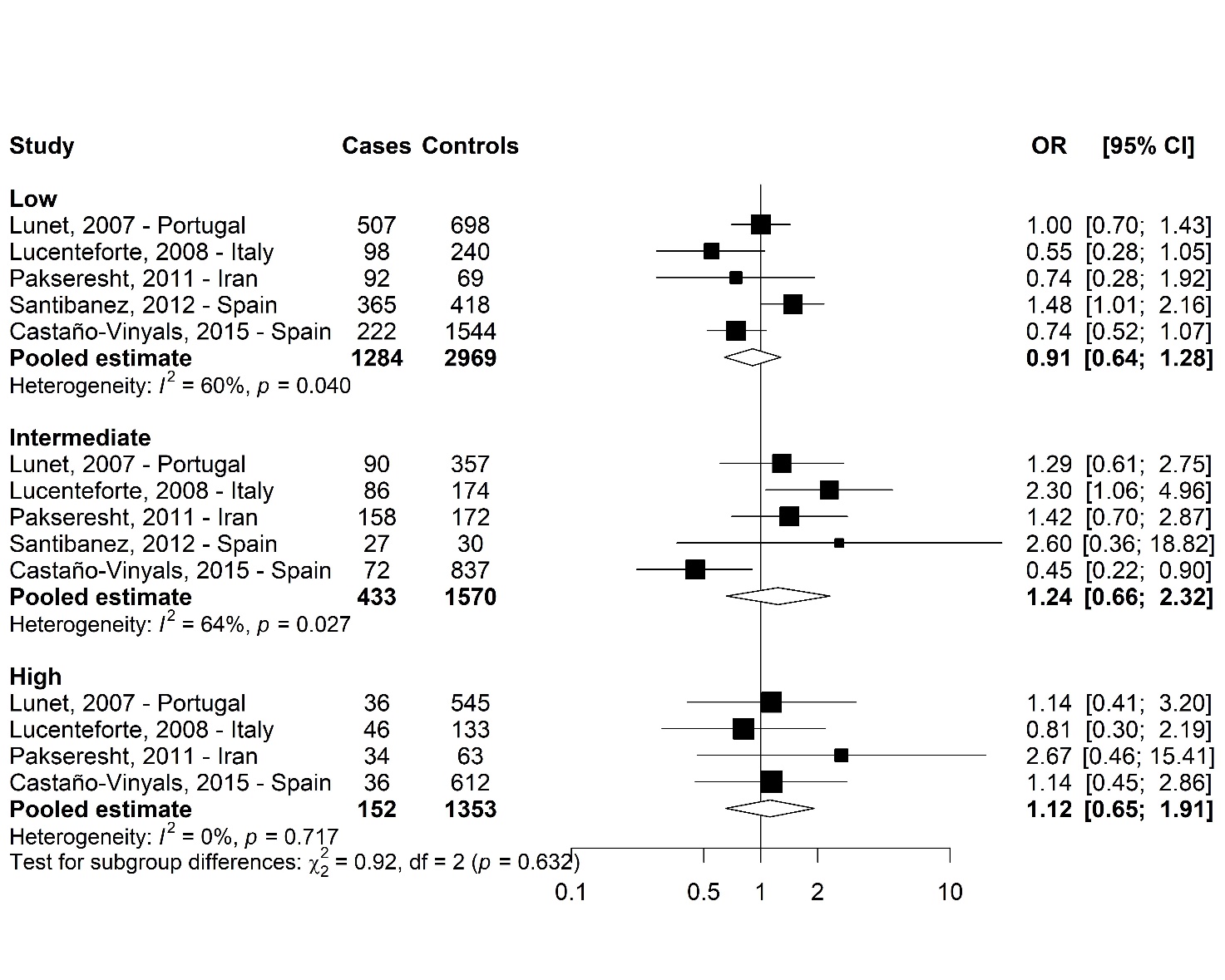


^a^ Estimated by two-stage meta-analysis using logistic regression models adjusted for sex, age, socioeconomic status, smoking status, family history of gastric cancer, Hp infection (when available), and total energy intake.

# **Supplementary Figure 5.** Forest plot for study-specific and pooled odds ratios (OR)^a^ with 95% confidence intervals (CI) of gastric cancer for the highest versus the lowest tertile of vitamin D intake, according to smoking status.

**
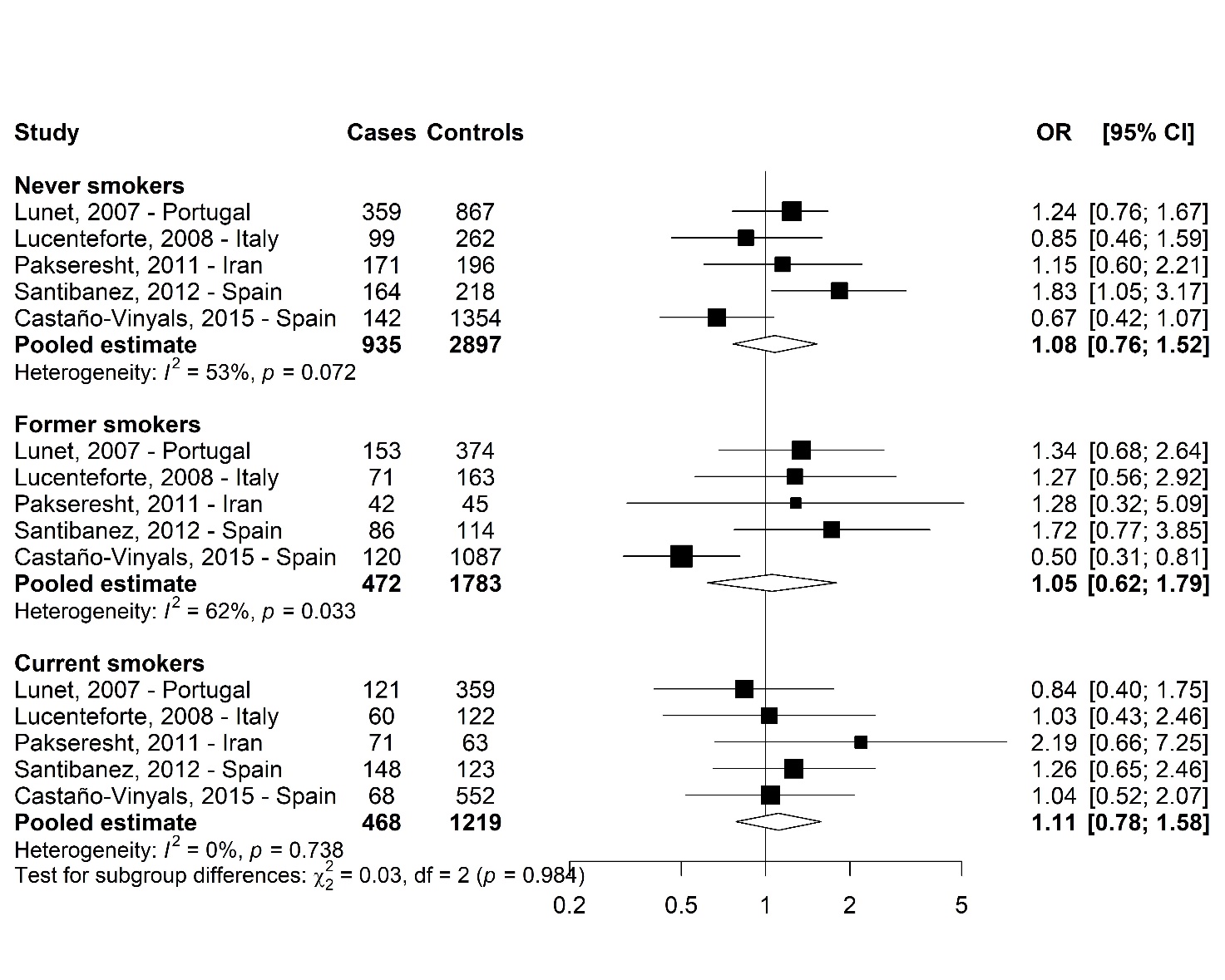
**

^a^ Estimated by two-stage meta-analysis using logistic regression models adjusted for sex, age, socioeconomic status, smoking status, family history of gastric cancer, *Hp* infection (when available), and total energy intake.

# **Supplementary Figure 6.** Forest plot for study-specific and pooled odds ratios (OR)^a^ with 95% confidence intervals (CI) of gastric cancer for the highest versus the lowest tertile of vitamin D intake, according to alcohol consumption.

**
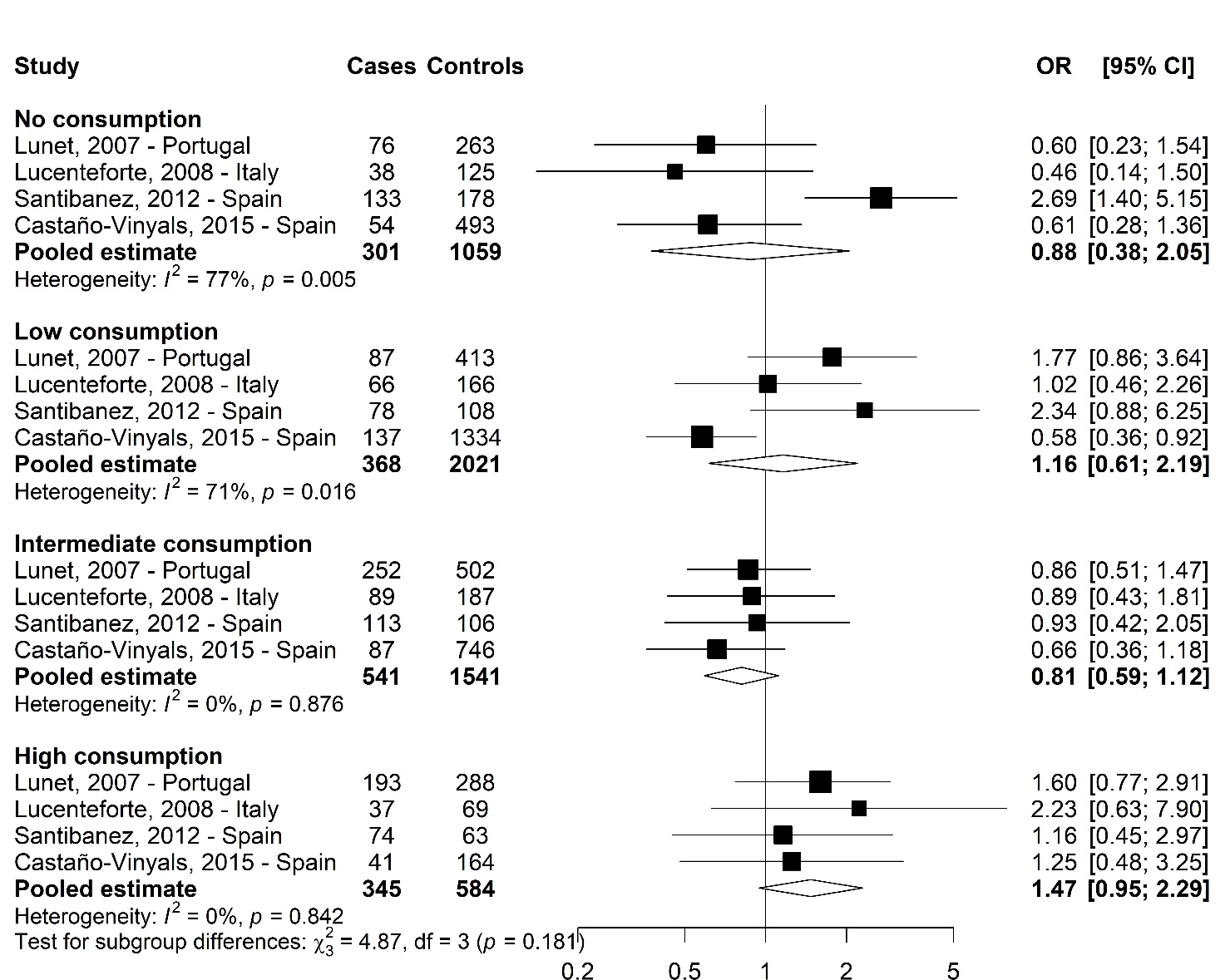
**

^a^ Estimated by two-stage meta-analysis using logistic regression models adjusted for sex, age, socioeconomic status, smoking status, family history of gastric cancer, *Hp* infection (when available), and total energy intake.

# **Supplementary Figure 7.** Forest plot for study-specific and pooled odds ratios (OR)^a^ with 95% confidence intervals (CI) of gastric cancer for the highest versus the lowest tertile of vitamin D intake, according to vegetables and fruits intake consumption.

**
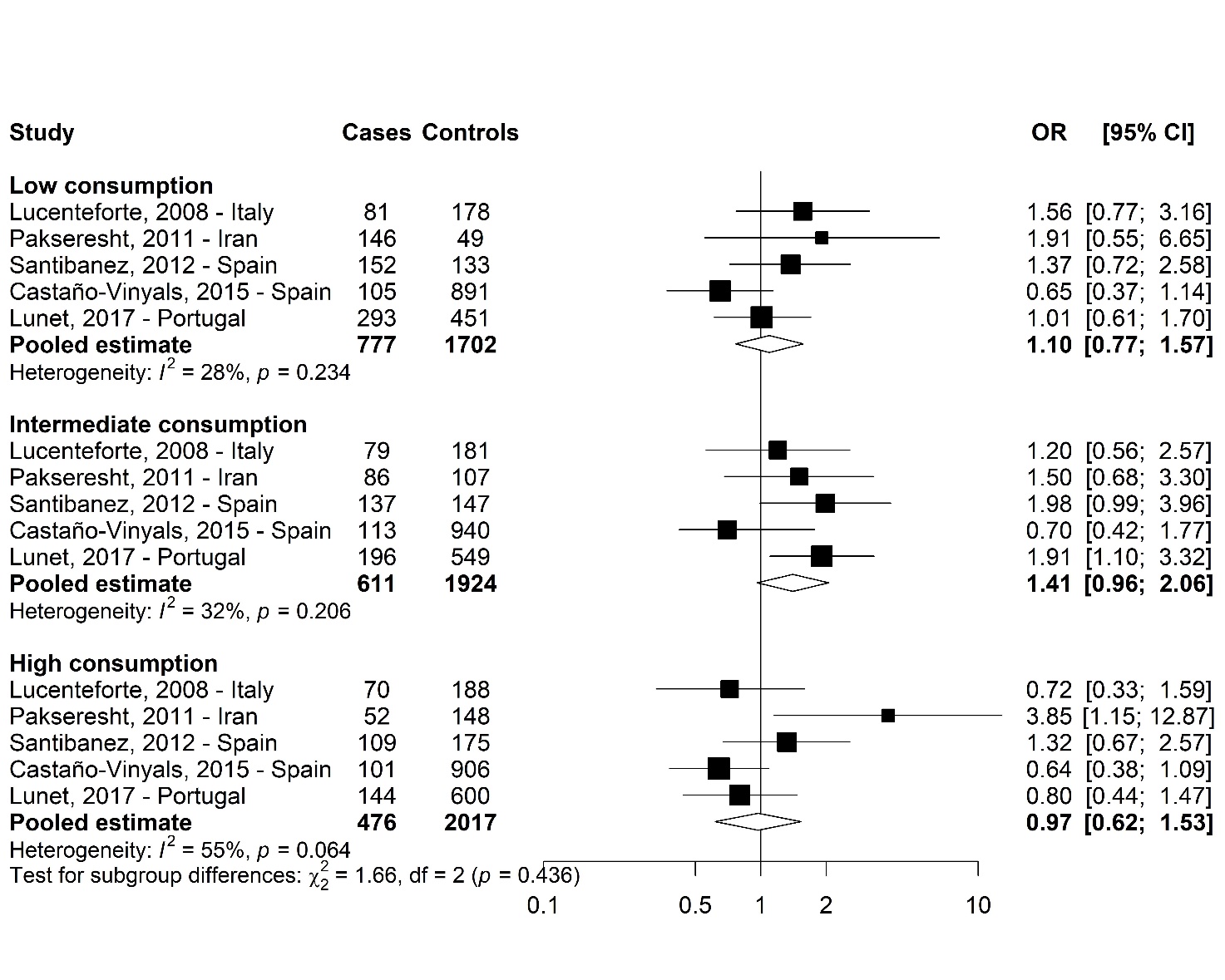
** ^a^ Estimated by two-stage meta-analysis using logistic regression models adjusted for sex, age, socioeconomic status, smoking status, family history of gastric cancer, *Hp* infection (when available), and total energy intake.
